# Lipid Droplets: A Key Cellular Organelle Associated with Cancer Cell Survival under Normoxia and Hypoxia

**DOI:** 10.3390/ijms17091430

**Published:** 2016-08-31

**Authors:** Shiro Koizume, Yohei Miyagi

**Affiliations:** Molecular Pathology and Genetics Division, Kanagawa Cancer Center Research Institute, 2-3-2 Nakao, Asahi-ku, Yokohama 241-8515, Japan; miyagi@gancen.asahi.yokohama.jp

**Keywords:** lipid droplets, cancer, normoxia, hypoxia, stress response

## Abstract

The Warburg effect describes the phenomenon by which cancer cells obtain energy from glycolysis even under normoxic (O_2_-sufficient) conditions. Tumor tissues are generally exposed to hypoxia owing to inefficient and aberrant vasculature. Cancer cells have multiple molecular mechanisms to adapt to such stress conditions by reprogramming the cellular metabolism. Hypoxia-inducible factors are major transcription factors induced in cancer cells in response to hypoxia that contribute to the metabolic changes. In addition, cancer cells within hypoxic tumor areas have reduced access to serum components such as nutrients and lipids. However, the effect of such serum factor deprivation on cancer cell biology in the context of tumor hypoxia is not fully understood. Cancer cells are lipid-rich under normoxia and hypoxia, leading to the increased generation of a cellular organelle, the lipid droplet (LD). In recent years, the LD-mediated stress response mechanisms of cancer cells have been revealed. This review focuses on the production and functions of LDs in various types of cancer cells in relation to the associated cellular environment factors including tissue oxygenation status and metabolic mechanisms. This information will contribute to the current understanding of how cancer cells adapt to diverse tumor environments to promote their survival.

## 1. Introduction

**Figure 1:**
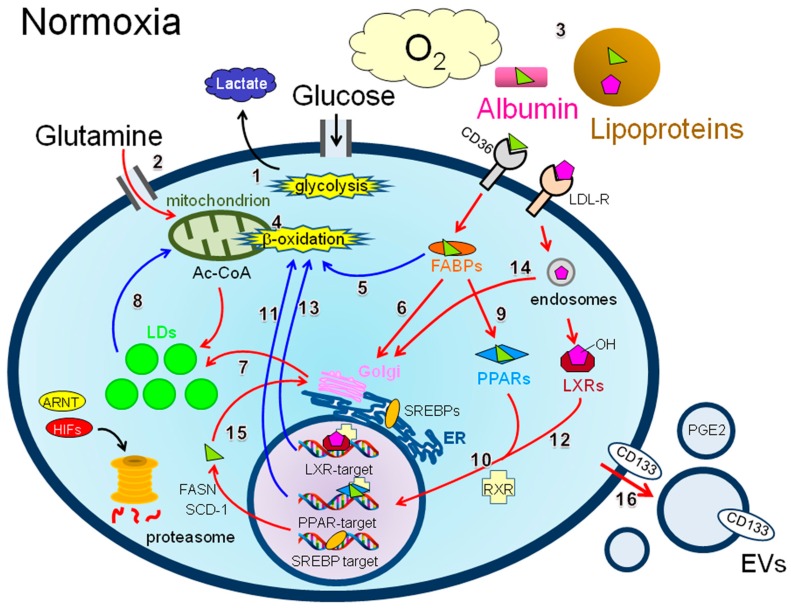
Schematic of the possible metabolic routes associated with lipid droplet (LD) synthesis in cancer cells exposed to normoxic conditions. Under normoxia, cancer cells are expected to have easy access to serum components. Such factors (O_2_, glucose, glutamine, and lipids) are designated by a larger font size compared with that of lactate. Major metabolic energy sources (glycolysis and β-oxidation) are depicted with star bursts. Metabolic routes (1–16) possibly associated with LD synthesis and β-oxidation are designated with red and blue arrows, respectively. Other routes are shown in black arrows. The abbreviations used are as follows: LDs = lipid droplets; ER = endoplasmic reticulum; HIFs = hypoxia inducible factors; ARNT = arylhydrocarbon receptor nuclear translocator; Ac-CoA = acetyl coenzyme A; LCFAs = long chain fatty acids; FABPs = fatty acid binding proteins; PPARs = peroxisome proliferator-activated receptors; LXRs = liver X receptors; SREBPs = sterol regulatory element binding proteins; RXR = retinoid X receptor; FASN = fatty acid synthase; SCD-1 = stearoyl CoA desaturase-1; LDL-R = low-density lipoprotein receptor; EVs = extracellular vesicles. The symbols used are as follows: 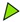: LCFAs, 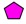: cholesterol, 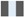: transporter, 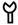: receptor.

Unlike normal cells, which mainly generate energy (adenosine triphosphate: ATP) through mitochondrial oxidative phosphorylation, cancer cells mainly use glycolysis to generate ATP even under sufficiently oxygenated conditions (aerobic glycolysis, also known as the “Warburg effect”) [1]. Cancer tissues can be exposed to hypoxia because of aberrant tumor vascularization, which induces the transcription of hypoxia inducible factors (HIFs) including HIF-1α and HIF-2α [2,3]. This leads to favorable conditions for this characteristic metabolism [1]. HIF-1α binds arylhydrocarbon receptor nuclear translocator (ARNT) and activates not only genes that promote glucose consumption, such as the glucose transporter solute carrier family 2 member 1 (*SLC2A1*, also known as *GLUT1*) [4] and hexokinase 2 (*HK2*) [1], but also genes involved in oxidative phosphorylation such as pyruvate dehydrogenase kinase 1 (*PDK1*) [1]. Increased levels of the PDK1 protein inhibit pyruvate dehydrogenase (pyruvate dehydrogenase phosphatase catalytic subunit 1 (*PDP1*, also known as *PDH*)) gene activity [1]. This inhibitory process leads to the suppression of acetyl-CoA production required for mitochondrial ATP production and de novo synthesis of lipids in the cytosol [4,5], thereby promoting cytosolic glycolysis to facilitate lactate secretion (Figure 1, route 1). Glutamine is another important energy source for cancer cells when glucose availability is limited [4,5] (Figure 1, route 2).

As in the case of glucose and glutamine, lipids [long chain fatty acids (LCFAs) and cholesterols] can be taken up from the bloodstream [6,7]. Normal cells tend to obtain LCFAs and cholesterols from the blood plasma via receptor proteins associated with the plasma membrane (Figure 1). Normal cells tend to rely on this exogenous source of lipids, although autonomous lipid production is also possible [8]. These exogenous source-derived lipids can be used for energy production, and the formation of biological membranes and other bioactive molecules [8]. Under physiological conditions, cellular lipid levels are maintained by a balance among uptake, de novo synthesis, consumption, and storage to maintain appropriate cellular functioning. Lipid droplets (LDs) are key organelles that function as storage of cellular surplus of lipid molecules in esterified form [9,10,11,12]. For example, adipose tissue is LD-rich and can store a large amount of LDs under physiological conditions [13].

Cancer cells may require large amounts of lipids for the synthesis of biomass and building blocks for lipid-derived bioactive molecules necessary for cellular membrane generation to maintain high cell proliferation levels [4,8]. Indeed, recent studies showed that cancer cells have a high lipid content and their high rate of proliferation is maintained by LCFA catabolism [14,15,16]. This characteristic lipid acquisition could be enhanced in cancer cells in response to hypoxia. A variety of LD functions are active in hypoxic cancer cells, and their activity is tightly controlled by various proteins via hypoxia-inducible transcriptional activation mechanisms. The characteristics of lipid metabolism and its importance in cancer cell biology have been widely reviewed in recent years [8,17,18,19,20,21,22]. The biology of LDs in normal (non-cancer) cells has also been extensively reviewed [10,11,12,23,24,25,26]. The aim of this review was to focus on how LDs are synthesized and function in various types of cancer cells in relation to cellular environmental factors including the cellular oxygenation status.

## 2. Lipid Droplets: Origin and Components

LDs are cytoplasmic organelles responsible for storing excess cellular lipids. LDs are composed of core lipid components, surrounded by an amphipathic lipid layer with various proteins [9,10,11,12]. Lipids are stored in LDs as neutral lipids, namely free fatty acids and cholesterols that are enzymatically converted to triacylglycerol (TAG) and cholesteryl esters (CEs), respectively, and then incorporated into LDs. LDs are generated at and derived from the endoplasmic reticulum (ER) and/or Golgi apparatus, given that many LD proteins such as perilipin and caveolin are associated with resident proteins of these organelle [9,10,25]. The size of LDs can vary and becomes larger through the fusion of smaller LDs as their generation proceeds [9,25]. Free LCFAs and cholesterols are enzymatically released from LDs and used as needed. The components of LDs can also vary depending on cell type. For example, adipocyte LDs are TAG rich, while CEs are primarily abundant in macrophage LDs [27]. LD components are heterogeneous and vary according to cancer cell type and the tumor microenvironment. Indeed, the composition of saturated-LCFA esters [28], unsaturated-LCFA esters [29], and CEs [30] changes depending on cancer cell type.

## 3. Exogenous and Endogenous Sources of LCFAs and Cholesterol

Normal and cancer cells can be supplied with LCFAs and cholesterols via exogenous sources in the bloodstream [31,32]. Various saturated or unsaturated LCFAs such as linoleic and oleic acid are important sources for LD formation. LCFAs are major components of serum lipids. Owing to their very low solubility in aqueous solutions, their free form can circulate in the bloodstream in a complex with albumin [33] (Figure 1, number 3). LCFAs can also exist in the blood as neutral lipids, namely their esterified form. LCFA esters are converted to TAG by sequential enzymatic steps initiated by glycerol-3-phosphate acyltransferase and incorporated into large lipoprotein complexes such as very low density lipoprotein and chylomicrons synthesized in the liver and intestinal cells (Figure 1, number 3).

Cholesterols from the diet or de novo synthesis in the liver are another important plasma lipid and are also water insoluble, similar to LCFAs. Lipoproteins serve as vehicles for cholesterols in the blood [34]. Cholesterols are converted to CEs by acyl-CoA cholesterol acyltransferases (ACATs) [34] and lecithin cholesterol acyltransferase. They are incorporated into the core of large lipoproteins, including low-density lipoproteins (LDLs) and high-density lipoproteins, in which TAGs composed of LCFAs are the major neutral lipids. LDLs are also enriched in CEs [34]. These large lipoprotein complexes are synthesized in the ER–Golgi system (Figure 1).

Cancer tissues are generally hypovascular, and therefore show poor acquisition of serum components including lipids. Cancer cells do not necessarily depend on exogenous lipids. Recent studies revealed that cancer cells can autonomously synthesize LCFAs and cholesterol via the same metabolic pathways that are active in normal cells [8] and/or aberrant metabolic pathways characteristic of cancer cells as outlined below.

## 4. Molecular Mechanisms Associated with the Biosynthesis of LDs in Cancer Cells

### 4.1. Decomposition of LDs through β-Oxidation

Cancer cells within a normoxic tumor environment should have access to serum components. Cancer cells tend to produce ATP through the activation of glycolysis rather than mitochondrial oxidative phosphorylation. Amino acids such as glutamine can also be used as an energy source in the mitochondrion. This process can be affected by growth factor-mediated cellular signaling [35].

In the healthy body, mitochondrial fatty acid oxidation (β-oxidation) (Figure 1, number 4) is active in limited organs, such as skeletal muscle [36], the heart [36], and the colon [37]. However, certain cancer cells such as prostate cancer cells [38], ovarian cancer cells [14], cancerous colonocytes [37], and breast cancer cells [39] use this mechanism as a major energy source despite the fact that β-oxidation represents an unfavorable metabolism given the predominance of the Warburg effect [1,4]. In addition, cancer cells should have access to exogenous LCFAs within well-vascularized tumor environments (Figure 1, number 3). Esterified LCFAs can be taken up into cancer cells from lipoproteins such as LDL. The non-esterified (free) form of LCFAs, which is bound by albumin, can be actively transported to the cytoplasm via the cell surface fatty acid transporter molecule CD36 (also known as fatty acid translocase or FAT) [40,41] (Figure 1). Passive transport (simple diffusion) is also possible [40]. LCFAs bind fatty acid binding proteins (FABPs) in the cytoplasm [42] (Figure 1) and are then transported to mitochondria to undergo β-oxidation (Figure 1, route 5) or incorporated into LDs in their esterified form (Figure 1, route 6 to 7). LCFAs can be used for β-oxidation by lipolysis of esterified LCFAs within LDs (Figure 1, route 8) and play primary roles in the pathogenesis of melanoma and ovarian and breast cancers [43].

Furthermore, the LCFA-FABP complex can traffic another metabolic route via peroxisome proliferation activator receptors (PPARs) (Figure 1, route 9), transcription factors known as one of the nuclear receptor families [41]. The PPAR-LCFA complex binds retinoid X receptor and then activates PPAR target genes [42] (Figure 1, routes 9 to 10). There have been experimental data showing that PPARs could facilitate β-oxidation in cancer cells. For example, PPARα can promote β-oxidation in liver cancer cells [44] (Figure 1, routes 9 to 11).

Cholesterols also play a role in the regulation of β-oxidation in cancer cells. Oxidized cholesterol derivatives bind the liver X receptor in the cytoplasm and then associate with retinoid X receptor (Figure 1, route 12) as in the case of PPARs. Liver X receptor target genes potentially control β-oxidation in cancer cells (Figure 1, routes 12 to 13). Indeed, cellular glucose uptake and LCFA oxidation are affected by this transcriptional mechanism in some cancer cells [45].

### 4.2. Production of LDs under Normoxia

A number of studies showed that cancer cells cultured under normoxia contain large amounts of LDs. Accumulating experimental evidence indicates that in some cancer types, such as renal clear cell carcinoma (RCC), cells are particularly lipid-rich when exposed to normoxic conditions. In this section, the cellular factors responsible for this normoxic LD synthesis and associated malignant phenotypes will be described with regard to several specific cancer types.

#### 4.2.1. Renal Clear Cell Carcinoma

The renal parenchyma is poorly oxygenated compared with the renal cortex, although the kidney is recognized as a well-perfused organ in the body [46]. However, because RCC cells are well vascularized, this cancer type should be less hypoxic than other typical tumors. Under normoxia, HIFs are immediately degraded by the proteasome (Figure 1). However, in most RCC cells, HIFs are constitutively expressed irrespective of oxygenation status because pVHL, an E3-ubiquitin ligase complex component [47,48], is not active, leading to the blockade of proteasomal degradation. Moreover, RCC is associated with an aberrant lipid metabolism including increased lipid storage [48]. Thus, it is possible that HIFs contribute to LD synthesis in RCC cells under conditions of normoxia, and indeed this is true. However, several lines of evidence indicate that HIF-independent mechanisms can also contribute to LD synthesis in RCC cells, as described below.

#### 4.2.2. HIF-Dependent Mechanisms of LD Synthesis in RCC Cells

**Figure 2:**
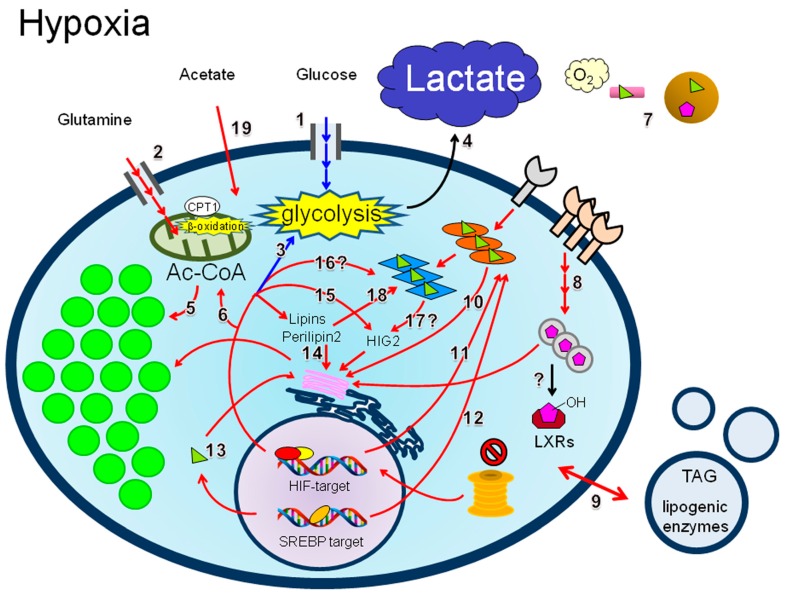
Schematic of the possible metabolic routes associated with LD synthesis in cancer cells exposed to O_2_-deficient conditions. Under hypoxia, cancer cells are expected to have restricted access to serum components. Cancer cells are also expected to secrete high levels of lactate under hypoxia. Serum components and lactate are designated with small and large font sizes, respectively. Under hypoxia, glycolysis and β-oxidation should be accelerated and suppressed, respectively. Accordingly, facilitated glycolysis and inactivated fatty acid oxidation are represented by large and small font sizes, respectively. Metabolic routes (1–19) possibly associated with LD synthesis and glycolysis are designated with red and blue arrows, respectively. Other routes are shown in black arrows. The abbreviations used are as follows: CPT1 = carnitine palmitoyltransferase 1; HIG2 = hypoxia inducible protein 2; TAG = triacylglycerol. The symbol “?” is indicative of potential contribution in cancer cells.

Cholesterol uptake by RCC cells can be enhanced by the upregulation of low-density lipoprotein receptor (LDL-R) (Figure 1), resulting in the accumulation of LDs [49] via endosomal cholesterol trafficking (Figure 1, route 14). RNA interference experiments revealed that this LDL-R upregulation is HIF-1α dependent. The formation of LDs mediated by increased cholesterol uptake contributes to the RCC phenotype [49]. Hypoxia-inducible protein 2 (HIG2) is highly expressed in RCC tissues and cells even under normoxic culture conditions [50] (Figure 2). The *HILPDA* gene encoding HIG2 is a target gene of HIF-1α [50]. HIG2 is an LD protein that plays an important role in LD production [51]. HIG2 expression levels and patterns in RCC tissues are consistent with those of HIF-1α, implying that the HIF-1α–HIG2 pathway is significant for LD production in RCC cells. The perilipin 2 protein is another example of a HIF-driven LD protein associated with RCC [52]. HIF-2α is responsible for the induction of the *PLIN2* gene, which encodes perilipin 2 and contributes to high LD synthesis in RCC cells.

#### 4.2.3. HIF-Independent Mechanisms of LD Synthesis in RCC Cells

Sterol regulatory element binding proteins (SREBP-1 and SREBP-2) are major transcription factors owing to the production of LDs via de novo LCFA synthesis (Figure 1, route 15 to 7) [8,53]. The immature form of SREBPs is present in the ER [48]. These transcription factors undergo sequential enzymatic cleavage when the exogenous cholesterol supply is limited, leading to the transport of the mature active form of SREBPs into the nucleus [48,53]. Thus, the activity of SREBPs is expected to be relatively low in normoxic RCC cells. However, a study showed high activity of SREBPs in RCC cells via the TRC8 protein [48]. SREBPs mediate the activation of multiple genes by binding to sterol regulatory elements within the regulatory regions of genes such as *FASN*, which encodes fatty acid synthase (FASN), and *SCD*, which encodes stearoyl CoA desaturase-1 (SCD-1) and is involved in LD formation in cancer cells [48] (Figure 1). SCD-1 is important for the viability of RCC cells, and pharmacological inhibition of this enzyme results in apoptotic cell death [54].

RCC cells show increased activity of ACAT-1 followed by an increase in the cellular level of esterified cholesterols [48,53]. However, there is no evidence that this ACAT increase is dependent on HIFs and SREBPs [48]. A recent study showed that autophagy plays an important role in LD formation in RCC cells [55]. The autophagy protein MAP1S negatively regulates LD synthesis and thereby suppresses the malignancy of RCC. This implies a role of the LD as a promoter of RCC aggressiveness. In addition, there is no evidence supporting the HIF dependence of MAP1S expression.

#### 4.2.4. Prostate Cancer

Prostate cancers (PCs) are highly dependent on lipid metabolism to express their malignant phenotypes [56,57]. To date, various phenotypes such as cell survival and cell migration were reported to be associated with LD formation in PC cells [56,57]. SREBP-1 is overexpressed in PC tissues [56], and this promotes the expression of the malignant phenotypes of PC cells cultured under normoxia in association with NADPH oxidase 5 expression, followed by the generation of reactive oxygen species (ROS) [56]. Also, p300 acetyltransferase can augment PC growth via FASN expression [57]. In addition, PCs are categorized as hormone (androgen) dependent or refractory cancer types. The expression of the androgen receptor (AR) is upregulated in response to SREBP-1 overexpression in PC cells, indicating the activation of the AR signaling pathway [56]. A recent Raman spectroscopy analysis revealed that the amount of LDs increases in response to androgen treatment [28].

As in the case of RCC, LD formation in PCs can be regulated by autophagy. LDs in PCs are degraded by this intracellular degradation mechanism during androgen deprivation, resulting in the promotion of cell growth [58,59]. Thus, it is possible that key phenotypes of androgen-sensitive PCs are mediated by an autophagy-LD metabolic pathway. In addition, oncogenic events can contribute to the aggressiveness of PC by promoting lipid uptake. The loss of the tumor suppressor gene *PTEN* followed by the activation of the PI3K-Akt-SREBP-1 axis increases LDL-mediated uptake of polyunsaturated fatty acids (PUFA) and cholesterol [30]. LD formation induced by increased lipid levels seems advantageous for PC growth [30].

#### 4.2.5. Breast Cancer

Breast cancer (BrC) is another malignancy that can be associated with high cytoplasmic LD content. This may be related to the fact that primary BrC tissues are within the mammary gland, which is rich in adipocytes. LD formation in BrC cells may be associated with the presence of hormone (estrogen and/or progesterone) receptors on the surface of cancer cells [60,61,62]. As in the case of PCs, a recent Raman spectroscopy analysis demonstrated that LDs increase in response to hormone treatment in BrC cells [28]. Progestin treatment may promote LD formation in BrC cells, and this is associated with SCD-1 expression [61], underscoring the importance of lipid desaturation by hormone-receptor mediated signaling pathways. This notion is supported by the fact that pharmacological inhibition of SCD-1 decreases the viability of BrC cells [61].

LD production in BrC cells can also be affected by hormone-independent mechanisms. Triple-negative BrC cells, which lack expression of hormone receptors and the cell surface HER2 protein, show high levels of expression of ACAT and LDL-R, which facilitate lipid uptake and cholesterol esterification [62,63]. The proliferation and motility of these BrC cells are enhanced by intracellular lipid storage, suggesting that LD formation is important for the expression of aggressive phenotypes. Other exogenous stimuli are reported to promote LD formation in BrC cells. Stimulation of cancer cells with insulin or unsaturated fatty acids can activate the expression of the ER protein ERLIN2 to promote LD synthesis in cells via de novo lipogenesis, thereby facilitating cell proliferation [64]. Group X secreted phospholipase A2 is an additional mediator of lipid metabolism in BrC cells [65]. Treatment of cancer cells with this lipolytic enzyme facilitates LD formation and subsequent β-oxidation to promote cell growth and survival [65]. These results indicate that LDs contribute to the expression of malignant phenotypes in BrC cells. In addition, treatment of BrC cells, including the BrC cell line MCF-7, with LCFA-rich albumin results in their transformation into adipocyte-like cells with abundant LDs [66]. Furthermore, PPARγ ligand treatment of MCF-7 cells resulted in an increase in LDs with a differentiation phenotype [67], suggesting that LCFA can be used for cancer differentiation therapy.

#### 4.2.6. Ovarian Cancer

Accumulating experimental evidence indicates that de novo lipid synthesis is accelerated in ovarian cancer cells [14,68,69,70,71]. The expression of FASN can be elevated in ovarian cancer cells in response to lysophosphatidic acid stimulation in association with increased cellular LD levels [69,70]. A major site of metastasis of ovarian cancer is the omentum, which is composed of adipose tissues [14]. Thus, ovarian cancer cells can directly acquire LCFAs from adipocytes within the omentum, leading to increased formation of LDs as an energy source [14]. As in the case of MCF-7 cells, the ovarian cancer cell line SKOV-3 acquires an adipocyte-like phenotype in response to specific unsaturated fatty acid treatment [66]. Recent reports show that cystathionine beta-synthase modulates the transcription factor Sp1 to increase the transcription of its target gene, *SREBF1* in ovarian cancer cells [71]. The SREBP-1 protein encoded by the *SREBF1* gene mediates LD synthesis, thereby enhancing cell proliferation, motility, and invasiveness [71]. A relationship between human sulfatase-1 (HSulf-1) expression and LD formation was also reported in conjunction with the prognosis of ovarian cancer patients [72]. Loss of HSulf-1 expression confers lipogenic phenotypes, with increased LD levels leading to the expression of malignant phenotypes of ovarian cancer cells [72]. However, HSulf-1 expression can result in lipolysis followed by β-oxidation in ovarian cancer cells [72]. These results suggest that HSulf-1 has a tumor-suppressing effect with the suppression of a lipogenic phenotype. Furthermore, immunohistochemistry experiments show that HIG2 is highly expressed in ovarian clear cell carcinoma tissues compared with RCC tissues [73].

#### 4.2.7. Colon Cancer

LDs are likely to play crucial roles in the progression of colorectal cancer (CRC) cells. Indeed, LDs are abundant in CRC tissues compared with normal tissue counterparts [74]. It has been reported that downregulation of the transcription factor FOXO3 correlates with suppression of the protein deacetylase, SIRT6, thereby increasing the cellular LD level in association with upregulation of the LD protein perilipin 2 [75]. This increased LD level associates with progression of CRC. A very recent study has shown that this FOXO3-SIRT6 axis-driven regulation of CRC cell proliferation via LD production can be controlled by cellular signaling associated with cell surface epidermal growth factor receptor (EGFR) [76]. It has recently been revealed by Raman spectroscopy that the cellular LD level associates with resistance of CRC cells to erlotinib, which targets EGFR [77]. It has also been reported that the cellular LD level may correlate with the stemness of CRC cells, as the expression of the stem cell marker, CD133, is increased, potentially leading to chemoresistance [78]. CD133 is a membrane glycoprotein that can be a component of membrane-derived small particles called extracellular vesicles (EVs) released from melanoma cells [79,80] (Figure 1, route 16). It has been shown that this EV secretion augments the metastatic potential of melanoma cells [79].

The progression of CRC is known to be dependent on exposure to lipid compounds such as prostaglandins (PGs) [81]. LDs in CRC cells can be a source of eicosanoids, precursors of PGs. It has been revealed that prostaglandin E_2_ (PGE2) synthesized in LDs promotes the progression of CRC [74,81]. Pharmacological inhibition of FASN blocks this PGE2 synthesis, followed by repressed cell proliferation [74], indicating that de novo lipogenesis is involved in LD-mediated CRC progression. PGE2 can be secreted via EVs from cancer cells to promote cell invasiveness and immunosuppression [22] (Figure 1, route 16). This PGE2 production is enhanced by Ras-induced oncogenic transformation of intestinal epithelial cells [74]. In addition, increased FASN expression contributes to the malignancy of CRC via a different pathway. Enhanced LD formation associated with FASN expression can augment energy production by β-oxidation in CRC cells, thereby facilitating the survival and metastatic potential of CRC cells [82].

### 4.3. LD Synthesis under Hypoxia

Recent studies show that the LD content in cancer cells is increased in response to hypoxia (Figure 2) and plays a role in the biology of cancer cells, as described later. In normal cells under normoxic conditions, de novo fatty acid synthesis is mediated by pyruvate production, followed by the transport of mitochondrial Ac-CoA to the cytosol via a citrate shuttle mechanism [4]. However, this should be a minor process in cancer cells because of the Warburg effect. Moreover, the external supply of glucose and glutamine to cancer tissues is limited under hypoxia (Figure 2, routes 1 and 2 designated with smaller font size). However, the transport of glucose and glutamine [83,84] can be enhanced (Figure 2, routes 1 and 2, designated by multiple arrows) when cells are exposed to hypoxia [1,4], as the proteins involved in these metabolic processes are upregulated in response to HIF-1α expression. The enhanced glycolysis (Figure 2, routes 1 and 3) results in increased lactate secretion, followed by the generation of an acidic environment (Figure 2, route 4).

Accumulating evidence indicates that lipogenesis is increased in cancer cells. Recent studies suggest that cancer cells synthesize lipids via reductive glutamine metabolism under conditions of hypoxia [85,86,87] (Figure 2, route 5), which is independent of pyruvate-mediated Ac-CoA production in the cytosol. Indeed, cancer metabolism is dependent on glutamine metabolism via oncogenic c-Myc expression under normoxia [83]. Inducible glutamine metabolism via HIF-1α is also crucial for cancer cells exposed to hypoxia [84,87,88] (Figure 2, route 6). Furthermore, genes encoding lipogenic enzymes such as FASN and SCD-1 are upregulated in cancer cells in response to hypoxia [8]. The induction of gene transcription mediated by SREBP-1 activation is associated with the expression of HIFs [89,90]. Moreover, cancer cells can accelerate the synthesis of Ac-CoA (Figure 2, designated as larger font size), a substrate of FASN via reductive carboxylation in mitochondria under conditions of hypoxia [85,86]. Thus, the induction of transcription driven by hypoxia may contribute to a high LD content (Figure 2, route 5) in hypoxic cancer cells. Paradoxically, a recent proteomic analysis in a colorectal cancer cell line revealed that lipogenic enzymes are suppressed in a HIF-1α dependent manner, suggesting that HIF-1α does not necessarily facilitate lipid biosynthesis [91]. In the next section, molecular mechanisms associated with hypoxia-driven LD synthesis in cancer cells, especially in relation to lipid uptake, de novo synthesis, and β-oxidation will be described.

#### 4.3.1. Lipid Uptake

As in the case of nutrients such as glucose and glutamine, cancer tissues exposed to hypoxia are insufficiently supplied with serum lipids (Figure 2, number 7). Therefore, cancer cells develop mechanisms to enhance lipid uptake in response to hypoxia. The upregulation of cell surface receptors for plasma lipids is one strategy used by cancer cells to obtain cellular lipids under harsh hypoxia conditions (Figure 2, route 8). Indeed, the expression of LDL-R is upregulated in RCC cells under normoxia [49]. This LDL-R induction is expected to be enhanced during hypoxia, as this process is HIF-1α-dependent. In addition, studies using BrC cell lines revealed that the expression of LDL-R-related protein (LRP-1) is increased in cells during hypoxia and contributes to cell survival and metastasis [92]. Hypoxia also facilitates fatty acid uptake into cancer cells, although this is accompanied by the suppression of de novo lipogenesis [93]. This study showed that cancer cells scavenge unsaturated fatty acids from plasma lysophospholipids to increase the cellular lipid content, although the detailed mechanisms are unclear. This phenomenon can be recapitulated by oncogenic Ras activation in cancer cells under normoxia [93].

Cancer cells may develop alternative means to take up extracellular lipids. Cancer cells in tumors can acquire TAG via extracellular vesicles (EVs) secreted from the surrounding hypoxic cancer cells [94] (Figure 2, route 9). A recent study showed that de novo lipogenesis in PC cells is enhanced to produce TAG in response to hypoxia, and this autonomously synthesized TAG is secreted via EVs [94]. Accordingly, neighboring normoxic cancer cells become accessible to extracellular lipids via EV-mediated uptake of TAG (indicated by a bidirectional arrow in Figure 2, route 9).

Brain cancers such as astrocytomas and glioblastoma multiforme (GBM) are known to be hypoxic cancers [95,96]. In particular, GBM is a lethal brain disease, and tissues of this cancer type contain highly hypoxic tumor regions associated with a pathological structure called the pseudopalisade [95]. Highly hypoxic (near to necrotic) glioblastoma regions contain a large amount of LDs [97]. Increased fatty acid trafficking through the upregulation of fatty acid binding proteins (FABPs) is another means to promote lipid uptake in cancer cells under hypoxia (Figure 2, route 10). A recent study in glioma and BrC cell lines revealed that the uptake of extracellular saturated and unsaturated fatty acids is promoted under hypoxia via the upregulation of FABP3 and FABP7, thereby leading to elevated LD formation [98]. The transcript levels of genes encoding these FABPs are increased in response to hypoxia in a HIF-1α-dependent manner [98] (Figure 2, route 11). In this case, de novo lipogenesis becomes suppressed in cancer cells [98]. However, in glioblastoma cells cultured under lipid-insufficient conditions, FABP-mediated uptake of lipids is predominantly regulated by SREBP-1 (Figure 2, route 12), although the detailed transcriptional regulation mechanisms remain unclear [99].

Lipid uptake by cancer cells may be affected by non-cancer cells within the tumor microenvironment. As described above, invading ovarian cancer tissues are surrounded by adipose tissues when in the omentum and can acquire LCFAs from adipocytes as the energy source. Lipid synthesis in adipocytes is increased by exposure to EVs (exosomes) produced from neighboring adipocytes [100]. This is owing to the acquisition of lipogenic enzymes enriched in the EVs [100]. Thus, adipocytes with increased lipid content may be another lipid source for ovarian cancer cells. Furthermore, LDs are increased in bone marrow-derived mesenchymal stem cells exposed to severe hypoxia, resulting in the differentiation of these cells into adipocytes in a HIF-1α-dependent manner [101]. Immune cells are also components of the tumor microenvironment [102]. Hypoxia is associated with the activation of immune responses in cancer tissues [102]. Lipid uptake by macrophages is increased in response to hypoxia in atherosclerosis [103], suggesting that hypoxia-driven uptake of extracellular lipids plays a role in tumor biology.

#### 4.3.2. De Novo Lipogenesis

Biogenesis is another route for LD increase in cancer cells. Lipogenic genes are upregulated in response to HIF-1α expression, suggesting that de novo lipogenesis can be enhanced in cancer cells under hypoxia (Figure 2, route 13). Indeed, the *SREBF* gene encoding SREBP-1 can be a target of HIF-1α [89]. Furthermore, the *SCD* gene, a target of SREBP-1, can also be a target for HIF-2α [91]. However, hypoxia is not necessarily favorable for LD synthesis given that the reaction catalyzed by SCD-1 is an O_2_-consuming step [21,104]. Recent studies using glioblastoma models showed that LD production in cancer cells is dependent on the hypoxia-driven uptake of serum lipids but not on de novo lipogenesis [98]. In addition, lipid biosynthesis in glioblastoma cells presumably associated with LD formation is mainly regulated by de novo fatty acid synthesis, as SREBP-1 is activated when cells are starved of both serum lipid and O_2_ [99]. In this case, the expression of FABPs is also increased in a SREBP-1-dependent manner, although the mechanisms underlying this transcriptional regulation remain obscure [99]. Thus, the potency of de novo lipogenesis under hypoxia in cancer cells may be dependent on tissue oxygenation conditions and/or cancer cell types with diverse levels and patterns of expression of genes responsible for lipid uptake and biosynthesis. In the next section, additional factors potentially affecting hypoxia-mediated LD synthesis in cancer cells will be described.

#### 4.3.3. Lipins

Lipins or phosphatidate phosphatases are present predominantly in adipocytes and catalyze the conversion of diacylglycerol into TAG. Lipins are also important for LD formation in cancer cells exposed to hypoxia (Figure 2, route 14). The expression level of lipin 1, but not that of lipin 2, is increased in the cervical cancer cell line HeLa and the hepatocellular carcinoma cell line Huh-7 in response to hypoxia, resulting in elevated production of LDs [105]. HIF-1α can directly bind to a distal gene regulatory region, thereby activating the promoter region of the lipin 1 (*LPIN1*) gene to increase cellular TAG levels [106]. This HIF-1α–lipin1 axis is inhibited by protein kinase CK1δ, as phosphorylation of HIF-1α by this kinase inhibits HIF-1α–ARNT complex formation [106], resulting in reduced LD levels in cancer cells. By contrast, HIF-2α plays a predominant role in lipin1-mediated LD formation in BrC cells [107]. Intriguingly, the effect of lipin1 on cellular LD production changes depending on specific hypoxia conditions. Exposure of cells to long-term hypoxia inactivates mTOR, leading to the translocation of cytoplasmic lipin 1 to the nucleus [107]. This nuclear lipin 1 inhibits SREBP-1, leading to decreased LD levels [107].

#### 4.3.4. Perilipin 2

Perilipin 2 is a LD membrane protein that is crucial for LD synthesis not only under normoxia, but also under conditions of hypoxia in cancer cells (Figure 2, route 14). The *PLIN2* gene encoding perilipin 2 (or adipophilin) is activated in multiple cancer cells [52,98]. Hypoxia-driven activation of *PLIN2* in glioblastoma cells was reported to be dependent on HIF-1α. However, *PLIN2* activation in RCC cells is predominantly regulated by HIF-2α under normoxia [52]. Thus, the HIF dependence of *PLIN2* expression can vary depending on cell type and/or cellular oxygenation conditions.

#### 4.3.5. HIG2

HIG2 is highly expressed in RCC cells even under normoxic conditions, as described above. This LD protein is upregulated in response to hypoxia in multiple cancer cells and contributes to the production of intact LDs [50]. The regulation of HIG2 expression at the transcriptional level is solely dependent on HIF-1α in cancer cells. Indeed, HIF-1α binds the promoter region of the *HILPDA* gene, which encodes HIG2, to activate transcription [51] (Figure 2, route 15). HIG2 is inducible in bone marrow-derived mesenchymal stem cells in response to hypoxia and is associated with their adipocytic differentiation [101]. Thus, HIG2 may play a role in LD formation in non-cancer cells within the tumor microenvironment.

#### 4.3.6. PPARs

PPARs may also contribute to LD synthesis under hypoxic tumor conditions. The *PPARG* gene encoding PPARγ is upregulated in response to hypoxia through HIF-1α induction in cardiac hypertrophy [108]. Indeed, HIF-1α binds to the promoter region of the *PPARG* gene to enhance TAG levels in cardiomyocytes by activating glycerol-3-phosphate dehydrogenase and glycerol phosphate acyltransferase, resulting in the pathological features characteristic of this disease [108]. However, whether this HIF-1α–PPAR pathway occurs in cancer cells remains unclear (Figure 2, route 16). In cancer, PPARα is upregulated at the transcriptional level in response to hypoxia in GBM cells [109,110]. This PPARα activation is associated with increased HIF-1α expression, number of peroxisomes, and LD levels [109,110]. The lipid components of LDs, including TAG and cholesterols, are increased in GBM cells in response to hypoxia [109,110]. In addition, PPARα can target the regulatory region of the *HILPDA* gene to enhance TAG storage in hepatocytes [111], although this PPAR-HIG2 axis has not been demonstrated in cancer cells (Figure 2, route 17). Lipins together with another coactivator, PGC-1α, could function as coactivators of PPAR gene expression in hepatocytes [112]. Thus, the lipin-PPAR interaction may contribute to LD synthesis in cancer cells exposed to hypoxia (Figure 2, route 18).

#### 4.3.7. Acetate

In addition to LCFAs and cholesterol, cancer cells can use plasma acetate as an exogenous source for lipid biosynthesis (Figure 2, route 19). A recent study showed that Ac-CoA synthase 2 (ACSS2), which catalyzes Ac-CoA production from acetate, ATP, and CoA, is upregulated at the transcriptional level in response to hypoxia in breast and prostate cancer cells [113]. This acetate-mediated Ac-CoA production in conjunction with increased FASN is expected to promote LD synthesis in cancer cells. Notably, this transcriptional upregulation under hypoxic conditions is synergistically enhanced when serum levels in the culture media are limited [113]. Activation of the *ACSS2* gene under conditions of limited oxygen and serum is dependent on SREBP-2 but not on SREBP-1 [113]. These results indicate that the consumption of serum acetate plays a critical role in the synthesis of cellular lipids, contributing to the development and progression of cancers under severe metabolic stress conditions.

#### 4.3.8. β-Oxidation and Hypoxia Reoxygenation

Generally, fatty acid oxidation should have a minor role in energy production in hypoxia-exposed cancer cells, given the low O_2_ availability and the Warburg effect [7] (Figure 2, designated as “β-oxidation” with smaller font size). Indeed, a recent study using hepatocellular carcinoma cells showed that HIF-1α suppresses β-oxidation under hypoxia [16]. HIF-1α inhibits the expression of acyl-CoA dehydrogenases by blocking the activation of the c-Myc-PGC-1α axis, leading to the suppression of β-oxidation and accumulation of LDs [16]. However, paradoxically, there is experimental evidence showing the importance of this LCFA catabolism in cancer cells exposed to hypoxia [114]. Carnitine palmitoyltransferase 1 (CPT1) is a key enzyme associated with the mitochondrial membrane and essential for LCFA uptake into this organelle to exert β-oxidation in cancer cells [12,13] (Figure 2). The growth of cancer cells in vivo is impaired by depletion of CPT1. CPT1 can be upregulated at the transcriptional level in cancer cells in response to hypoxia and functions to reduce LD levels in tumors [114], suggesting that the function of β-oxidation is retained in cancer cells under limited O_2_ supply. Hypoxic conditions in tumor regions are not constant and can vary according to various factors associated with tumor perfusion conditions [115]. As in intermittent hypoxia or cycling hypoxia, hypoxic tumor regions can be repeatedly exposed to reoxygenation [115]. Recent studies showed that the reoxygenation process followed by energy acquisition by β-oxidation is crucial for the growth of cancer cells exposed to hypoxia [94,98].

## 5. Function of LDs in Cancer Cells

As mentioned before in this review, LDs are associated with various malignant phenotypes. Accumulating experimental evidence supports the diverse roles of LDs in the adaptation of cancer cells to stress conditions, by which they contribute to the maintenance of homeostasis in cancer cells. The next section addresses such stress response mechanisms associated with LDs.

### 5.1. Roles of LDs in ER Homeostasis

**Figure 3:**
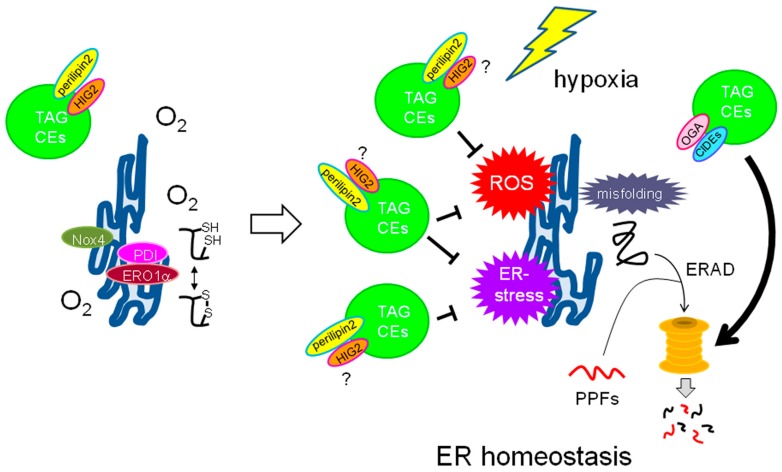
Potential effect of LDs on the ER-stress condition in cancer cells caused by O_2_ deficiency. Cancer cells sufficiently supplied with O_2_ show proper protein folding processes in the ER, including disulfide bond formation in association with multiple enzymatic activities as depicted. However, these processes may be dysregulated under hypoxia, thereby leading to redox imbalance. Accumulated misfolded proteins result in the ER-stress condition with generation of ROS. LDs are expected to suppress this toxic condition. Perilipin2 contributes to the maintenance of ER homeostasis. HIG2 is another LD membrane protein; however, its effect on ER homeostasis during hypoxia is currently unclear. OGA and Cidec potentially contribute to ER homeostasis by promoting ERAD. Enhanced degradation of PPFs via ERAD potentially contributes to survival of cancer cells exposed to hypoxia. The abbreviations used are as follows: Nox4 = NADPH oxidase 4; PDI = protein disulfide isomerase; ERO1α = ER oxidase 1α; CEs = cholesterol esters; ROS = reactive oxygen species; OGA = protein-*O*-linked *N*-acetyl-β-glucosaminidase; CIDEs = cell death-inducing DFF45-like effectors; ERAD = ER-associated degradation; PPFs = proteolytic protein fragments. The symbol “?” is indicative of potential contribution of HIG2. White, grey, and black bold arrows are indicative of transition from normoxia to hypoxia, protein degradation, and activation process, respectively.

Cancer cells need to synthesize high levels of biomass such as proteins to meet the demands associated with an increased cell proliferation rate [4,35]. Under such conditions, cancer cells tend to undergo “ER-stress,” which is characterized by defects in the proper folding of newly synthesized proteins in the ER owing to stresses such as protein overload, inappropriate glycosylation, and calcium imbalance [116,117] (Figure 3). ER stress is also induced in cancer cells via altered metabolism of membrane lipids [22]. ER-associated degradation (ERAD) is a cellular mechanism responsible for elimination of such aberrant proteins via the proteasome [116,117]. It has been shown that LDs could associate with the proteasome [118] and positively regulate its function via LD proteins such as protein-*O*-linked *N*-acetyl-β-d-glucosaminidase (OGA) in adipocytes [119], and cell death-inducing DFF45-like effector (CIDE) proteins in adipocytes and cancer cells [120,121,122], suggesting potential contribution of these proteins to maintaining ER homeostasis in cancer cells exposed to hypoxia (Figure 3). Prolonged and/or severe ER stress leads to programmed cell death (apoptosis). Cells adapt to these harmful cellular conditions by inducing an unfolded protein response in the presence of ER stress. Cancer cells under ER stress show abnormal ER morphology in response to limited serum and hypoxia, thereby promoting cell death and inducing the unfolded protein response [116,117]. However, this process can be prevented through the replenishment of unsaturated LCFAs rather than saturated LCFAs, indicating that the desaturation process of de novo lipogenesis under hypoxia is crucial for the survival of cancer cells [123,124]. This is consistent with the fact that SCD-1 is an O_2_-requiring enzyme and its activity is strongly repressed under hypoxia. In addition, LDs may contribute to ER homeostasis in cancer cells by ablation of proteolytic protein fragments (PPFs). It is known that PPFs are selectively degraded by proteasome according to N-end rule pathway [125,126]. Further, relationship between ER and N-end rule pathway has been documented [127,128]. Thus, it is likely that facilitation of proteasome function by LDs augments survival of cancer cells under hypoxia (Figure 3).

**Figure 4:**
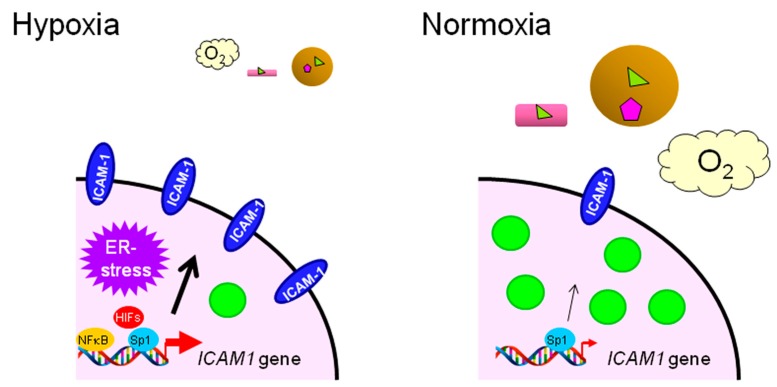
Model of *ICAM1* induction in ovarian cancer cells exposed to LCFA deficiency and hypoxia (left schematic). Expression of the *ICAM1* gene is robustly enhanced (a bold red bent arrow) under this stress condition, leading to high ICAM-1 expression on the cell surface (a black bold arrow). ER stress is also induced in cancer cells; however, it is not responsible for this transcriptional activation. In contrast, *ICAM1* expression is relatively low in cancer cells exposed to normoxia (right schematic). The LD level in these cells is relatively high owing to a sufficient LCFA supply.

By contrast, a recent study using ovarian cancer cells showed that the *ICAM1* gene, which encodes intercellular adhesion molecule-1 (ICAM-1), is synergistically activated when cells are exposed to both LCFA starvation and hypoxic conditions [129] (Figure 4). *ICAM1* expression associates with collaboration between the transcription factors, HIFs, Sp1 and NFκB [129] (Figure 4). The induced ICAM-1 protein is responsible for the survival of ovarian cancer cells by inhibiting apoptosis under severe hypoxic conditions that trigger ER stress [129] (Figure 4). However, *ICAM1* induction is independent of the stress response mechanism [129], suggesting that alternative cell survival mechanisms also exist. In vivo experiments showed that ICAM-1 is indeed strongly induced in severe hypoxic areas and is crucial for tumor growth. The hypoxic area was found to be poorly supplied with plasma LCFAs, followed by low cellular LD content [129]. Thus, it is feasible that cell survival mechanisms under ER stress-induced conditions vary depending on cell type and/or biological context.

Unsaturated LCFAs can reverse the cytotoxic effects associated with stress caused by a limited supply of both O_2_ and serum; however, a question arises regarding how unsaturated LCFAs are able to alleviate ER stress. A recent study by the same research group using RCC cells provided an explanation. HIF-2α expressed in RCC cells is responsible for the expression of the LD surface protein perilipin 2 (Figure 3) at the transcriptional level, which promotes the formation of LDs [52]. Loss of perilipin 2 and a reduced amount of LDs affect ER homeostasis [52], suggesting that LD synthesis associated with the HIF-2α–perilipin2 axis is significant for cancer cell survival via regulation of ER homeostasis.

### 5.2. Role of LDs as a ROS Scavenger

#### 5.2.1. ROS Generation in Hypoxic Cancer Cells

ROS are robustly produced in cancer cells with a reprogrammed metabolism. A major source of ROS in normal cells is mitochondrial oxidative phosphorylation [130]. Thus, it is likely that ROS production in cancer cells, which relies on the Warburg effect, is generally an unfavorable process. However, experimental evidence indicates that ROS can be released from mitochondria in hypoxia-exposed cells [130].

The ER can also be a source of ROS in cells exposed to various ER-stress-inducing conditions [130,131]. Protein folding associated with intramolecular disulfide bond formation is mediated by the interplay among ER-associated redox regulators such as protein disulfide isomerase (PDI), ER oxidase 1α (ERO1α), and NADPH oxidase 4 (Nox4) [127] (Figure 3). Protein misfolding associated with defects in this process may play a role in ROS production in cells during hypoxia-induced ER stress [131] (Figure 3). Thus, ROS production in cancer cells may be associated with ER stress in response to various metabolic stresses. Indeed, thalidomides can bind to LDs and induce ER stress to cause cell death [131]. This effect is enhanced when cancer cells are treated with certain PUFAs [131]. The hypoxia–reoxygenation process is a more likely candidate as a ROS source in cancer cells, as robust ROS synthesis occurs in cancer cells in response to the repeated sudden inflow of O_2_ into cancer tissues [130].

#### 5.2.2. LDs as Potential ROS Scavenging Organelles in Cancer Cells

Excessive ROS production in cells is harmful, as they damage cellular proteins, nucleic acids, and lipids. Cells have therefore developed antioxidant defense mechanisms associated with superoxide dismutase, glutathione peroxidase, and catalase to eliminate toxic oxygen derivatives [132]. Evidence indicates that LDs can function as ROS scavengers in cancer cells (Figure 3). De novo lipogenesis followed by the generation of the cellular membrane with saturated fatty acids is facilitated in cancer cells [133]. This membrane structure with its characteristic physical properties contributes to the resistance to lipid peroxidation [133]. A recent report revealed an antioxidant function of LDs using the *Drosophila* neural stem cell niche [132]. This study showed that hypoxia-driven production of LDs associated with PUFAs is increased in glial cells. These LDs function as an antioxidant to prevent the oxidation of cellular PUFAs, thereby facilitating *Drosophila* development [132]. It is unclear whether the same principle can be applied to cancer cells. Nevertheless, both saturated and unsaturated LCFAs are likely to play critical roles in preventing the cytotoxicity of ROS produced in response to hypoxia in cancer cells. Intriguingly, the role of LDs as a ROS scavenger described here is in contrast with the fact that ROS can function to promote the malignant phenotypes of prostate cancer cells [56].

### 5.3. Drug Resistance

The involvement of LDs in the drug resistance of cancer cells has become apparent. De novo lipogenesis rather than lipid uptake is associated with lipid saturation in the plasma membrane, resulting in chemoresistance of cancer cells [133]. Furthermore, it has been shown that lipid desaturation is critical for drug-resistance of various cancer cells [21]. A lipogenic enzyme, FASN, is important for drug resistance in various cancers [134,135], although the underlying mechanism remains obscure. The drug sequestration mechanism described below may be related with this phenotype.

As in the case of hypoxia exposure, cellular LD levels increase in response to drug treatment. A Raman spectroscopy analysis showed that LDs increase in hormone receptor-positive breast and prostate cancer cells in response to hormone treatment [28,60], suggesting a role for LDs in the resistance to hormone-related drugs. Other spectroscopic analyses showed that LDs are increased in various cancer cell types, such as breast, prostate, and ovarian cancer cells, and contribute to resistance to paclitaxel [136] and platinum agents [29,137,138]. LDs are likely to contribute to resistance of CRC cells to erlotinib [76,77]. The levels of LD components such as saturated LCFAs, unsaturated LCFAs, and CEs may affect this drug resistance. Although the detailed molecular mechanisms remain unclear, alterations in membrane fluidity owing to variations in the composition of these lipids may explain this characteristic [60,133]. Cancer stem cells may be involved in LD-associated drug resistance as expression of a stem cell marker CD133 correlates with LD level in CRC cells [78,139].

Another explanation for the role of LDs in promoting chemoresistance in cancer cells is drug sequestration. Carboxyesterase activity was shown to be suppressed in drug-resistant myeloid leukemia cells [140], and LDs are abundant in these cells. Aminopeptidase inhibitors, which are chemotherapeutic prodrugs, fail to be esterified upon uptake in drug-resistant cells, and are thereby sequestered in LDs [140]. The amino acids produced by the recovered aminopeptidase activity promote cancer cell proliferation via activation of mTOR [140]. A similar LD-mediated drug sequestration mechanism was proposed in docetaxel-resistant BrC cells [60].

A very recent study demonstrated that a bitter taste receptor, T2R38, is expressed in pancreatic cancer cells in association with LDs [141]. T2R38 functions as a transcription factor and enhances the expression of multi-drug resistance protein 1, which confers chemoresistance to cells [141]. However, the role of LDs in this process remains unclear. In addition, increased glucosylceramide levels and accumulation in LDs have been reported in multidrug-resistant cancer cells, suggesting that glucosylceramide is important for LD production and contributes to drug resistance [142].

## 6. Summary and Perspectives

As previously reported in the literature, lipid levels in cancer cells are generally high. In addition, desaturation of LCFAs plays pivotal roles in lipid-mediated cancer phenotypes. The absence of cellular lipids can induce the expression of genes encoding enzymes required for de novo lipogenesis to maintain proper cell function. Thus, potential therapeutic interventions in cancer based on targeting lipid biosynthesis have been explored. As described in this review, cellular lipids and their saturation status are associated with LD levels in cancer cells. Cellular LD levels fluctuate depending on lipogenesis, lipolysis, and lipid uptake. These processes can be enhanced or suppressed in response to hypoxia in cancer cells, and the resulting LDs are expected to play roles in adaptive responses to harmful conditions, such as ER stress associated with O_2_ insufficiency and drug toxicity. However, how cancer cells regulate these processes to generate LDs in response to stress conditions remains unclear. Moreover, ROS may function to promote or suppress malignancy in relation to LD biogenesis depending on cancer type, although their relative significance is unclear. Furthermore, it remains obscure how differences in tissue acidity within tumors as a result of a reprogrammed metabolism affect the cellular availability of plasma lipids. Improving our understanding of how cancer cells overcome such stress conditions in association with LD levels would facilitate the development of novel therapeutic strategies for the treatment of cancer.

In this review, it was also suggested that, in addition to O_2_ deficiency, hypoxic tumor areas also tend to be devoid of plasma lipids. It becomes apparent that transcriptional regulation mechanisms induced in response to lipid starvation other than those mediated by O_2_ deficiency play crucial roles in the adaptive response to severe tumor conditions. However, it is currently unclear which cancer cells show an inefficient supply of plasma lipids in response to diverse hypoxic tumor conditions. The poor vascularity in cancer tissues results in chronic O_2_ insufficiency, followed by limited gradients of molecules supplied from the blood. However, it is unclear to what extent serum components with different molecular characteristics diffuse into the same hypovascular cancer tissues. For example, are O_2_, glucose, and LCFAs complexed with high-molecular-weight albumin equivalent in terms of tissue diffusibility? In any case, cancer cells have diverse means to overcome the severe hypoxic tumor micro-environment in addition to those used to adapt to O_2_ deficiency via LD production. These mechanisms are likely to be context and/or cancer-type dependent. Further detailed investigation of the molecular mechanisms underlying LD-associated stress responses will improve our understanding of cancer cell survival and facilitate the development of promising strategies for cancer treatment.
